# *Plasmodium* infections and associated risk factors among parturients in Jawi district, northwest Ethiopia: a cross-sectional study

**DOI:** 10.1186/s12936-023-04803-z

**Published:** 2023-12-01

**Authors:** Zemenu Tamir, Abebe Animut, Sisay Dugassa, Mahlet Belachew, Adugna Abera, Aster Tsegaye, Berhanu Erko

**Affiliations:** 1 Department of Medical Laboratory Sciences, College of Health Sciences, Addis Ababa University, Addis Ababa, Ethiopia; 2 Aklilu Lemma Institute of Pathobiology, Addis Ababa University, Addis Ababa, Ethiopia; 3 Malaria and Neglected Tropical Diseases Research Team, Ethiopian Public Health Institute, Addis Ababa, Ethiopia

**Keywords:** *Plasmodium* infection, Placental malaria, Pregnancy, Parturient women, Risk factors, Jawi district, Ethiopia

## Abstract

**Background:**

Pregnant women have an increased risk of *Plasmodium* infections and disease. Malaria in pregnancy is a major public health problem in endemic areas. Assessment of the burden and risk factors of malaria in pregnancy across different malaria transmission settings is required to guide control strategies and for malaria elimination. Thus, the current study is generating such evidence from parturient women in northwest Ethiopia.

**Methods:**

A cross-sectional study was conducted among 526 pregnant women admitted to the delivery rooms of selected health facilities in Jawi district, northwest Ethiopia, between November 2021 and July 2022. Data on the socio-demographic, clinical, obstetric, and malaria prevention practices of pregnant women were collected using interviewer-administered questionnaires and from women’s treatment cards. Malaria was diagnosed by light microscopy, rapid diagnostic test, and multiplex real-time polymerase chain reaction. Risk factors for malaria were evaluated using bivariable and multivariable logistic regression models. A P-value of < 0.05 was considered statistically significant.

**Results:**

Among the examined parturient women, 14.3% (95% CI 11.4–17.5%) had *Plasmodium* infections. The prevalence of peripheral, placental, and congenital malaria was 12.2% (95% CI 9.5–15.3%), 10.9% (95% CI 8.2–14.1%), and 3.7% (95% CI 2.3–6.1%), respectively. About 90.6% of peripheral and 92% of placental *Plasmodium* infections were asymptomatic. *Plasmodium* infection at parturiency was independently predicted by maternal illiteracy (AOR = 2.03, 95% CI 1.11–3.74), primigravidity (AOR = 1.88, 95% CI 1.01–3.49), lack of antenatal care follow-up (AOR = 2.28, 95% CI 1.04–5.03), and history of symptomatic malaria during pregnancy (AOR = 4.2, 95% CI 2.32–7.59). Moreover, the blood group O phenotype was significantly associated with placental malaria among the primiparae.

**Conclusions:**

Overall, asymptomatic *Plasmodium* infections were prevalent among parturients in northwest Ethiopia. Maternal illiteracy, primigravidity, lack of antenatal care follow-up, and history of symptomatic malaria during pregnancy were the risk factors for malaria during parturiency. Thus, promotion of a healthy pregnancy through ANC follow-up, strengthening malaria prevention and control practices, and screening of malaria in asymptomatic pregnant women are suggested to reduce its burden in pregnancy.

## Background

Malaria remains a major public health and socioeconomic challenge in Africa, where it causes the highest proportions of global infections and deaths every year [1]. In Ethiopia, malaria due to *Plasmodium falciparum* and/or *Plasmodium vivax* occurs in about 68% of the landmass and threatens over 60% of the population [2]. The country contributed 1.7% of the global malaria cases and 1.5% of deaths in the year 2021 [1].

Pregnant women are more susceptible to malaria infection than non-pregnant women due to pregnancy-associated hormonal, nutritional, and immunological changes [3]. Malaria infection during pregnancy results in gestational malaria in the mother, placental malaria in the placenta, and congenital malaria in the newborn [4]. Placental malaria is attributed to the sequestration of infected erythrocytes in placental intervillous spaces, by binding to chondroitin sulfate A (CSA) receptors on the syncytiotrophoblast [5, 6], whereas congenital malaria is due to the transplacental transmission of the parasites from the mother to the fetus during pregnancy or delivery [7]. In the absence of pregnancy-specific interventions, about 45% of pregnant women and 41% of live births may experience malaria infections in Africa [8]. Although neglected, congenital malaria infection was about 6.9%, which showed great heterogeneity between unstable (16.8%) and stable (3.5%) malaria transmission areas [9].

In addition to the increased susceptibility, pregnant women and their newborns endure severe adverse outcomes associated with malaria, such as severe maternal morbidity, a high risk of maternal mortality, low birth weight, infant mortality, maternal anaemia, and congenital malaria [4, 10, 11]. However, the disease severity and adverse outcomes depend on the transmission intensity of the disease. In low and unstable transmission areas, infected pregnant women experience severe illness, which results in severe anaemia, miscarriage, stillbirth, and maternal death, particularly in young primigravid women [12]. In high and stable transmission settings, most infections remain asymptomatic but cause maternal anaemia, intrauterine growth restriction, and low birth weight, especially in older and multigravid women [13]. Such infections are mostly left untreated and serve as potential sources of new infections [14].

To reduce the burden of malaria, Ethiopia implements insecticide-treated bed nets (ITNs), indoor residual spray (IRS), and effective case management as core malaria prevention and control strategies [15, 16]. The prevention and control activities provided on the antenatal care (ANC) platform prioritize ITN provision and health education [17]. However, low levels of ANC follow-up and ITN utilization were reported among pregnant women in the country [18, 19].

On the other hand, malaria in pregnancy remains poorly studied in Ethiopia, particularly among parturient women. Previous studies on malaria among Ethiopian parturients reported prevalences ranging from 2.3% to 15.2% and identified the association of primigravidity with malaria [12, 20]. However, these studies were either conducted relatively on a small sample size in a single health facility and did not analyse independent risk factors other than observing simple associations [20, 21] or focused on comparing malaria burden in stable and unstable transmission sites [12]. Moreover, they used light microscopy as the only diagnostic tool to estimate malaria prevalence, which could compromise the findings. Thus, this study investigated the prevalence and associated risk factors of malaria among parturients in Jawi district, northwest Ethiopia, where malaria transmission is high [16].

## Methods

### Study design, setting and participants

**Fig. 1:**
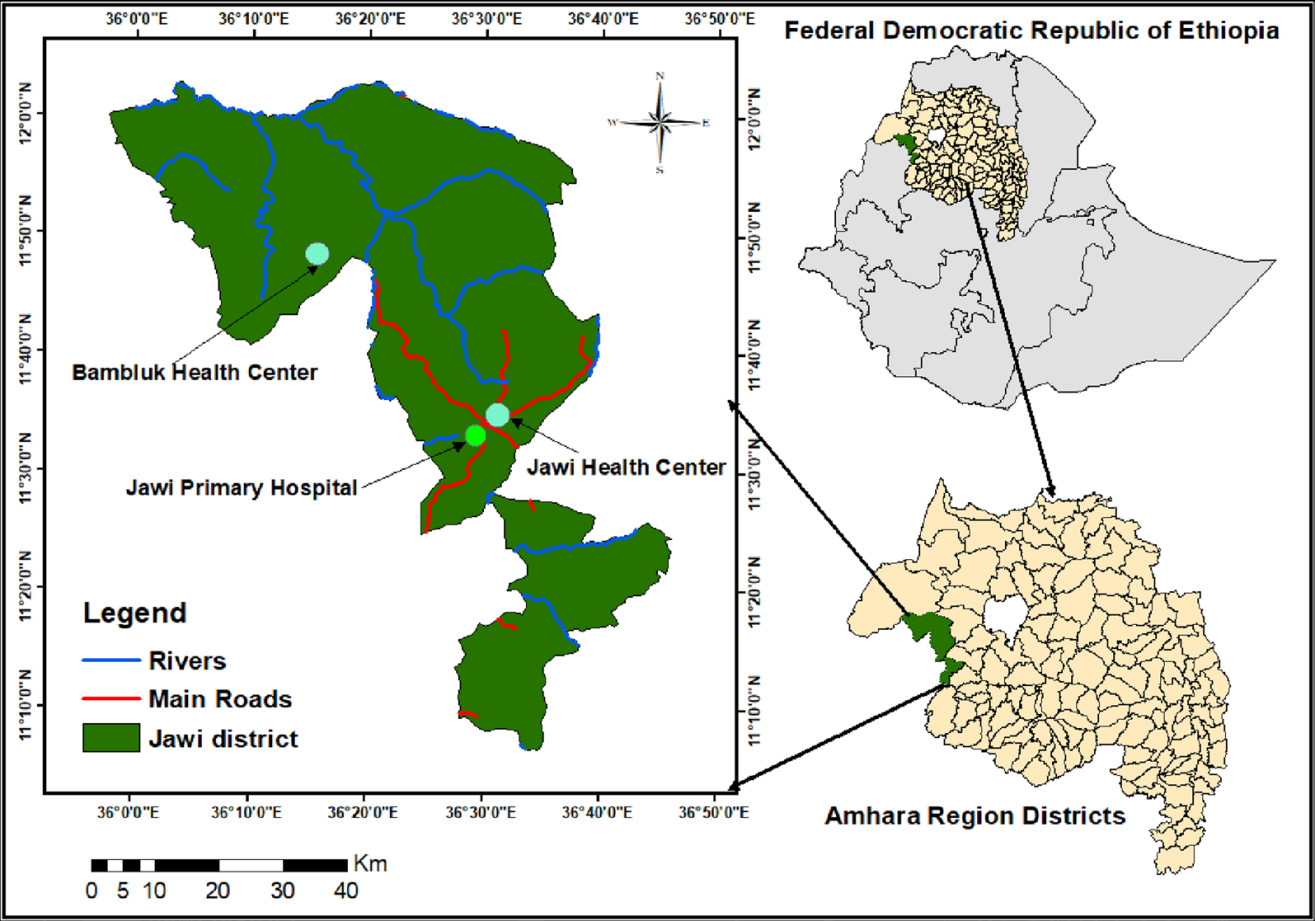
Map of Ethiopia showing the study areas

A health facility-based cross-sectional study was conducted among pregnant women admitted to the delivery rooms of Jawi Primary Hospital, Jawi Health Centre, and Bambluk Health Centre in Jawi district, Awi Zone, Amhara Regional State, northwest Ethiopia, for delivery or spontaneous abortion between November 2021 and July 2022 (Fig. 1). The study health facilities were selected based on the malaria transmission level along their vicinities. Jawi district had a mean annual rainfall of 1569.4 mm, a mean temperature of 18.2 to 32.4 °C, and an altitude range of 648 to 1300 m above sea level. Its population was 146364 in 2021 (Jawi District Administrative Office, unpublished document). It is one of the development corridors in Amhara Regional State where agricultural investments are practiced and seasonal migrant workers are common. The district is among the most malaria-endemic areas in the region, where year-round transmission occurs [22]. In 2021/2022, 25,906 malaria cases were reported (Jawi District Health Office malaria case report).

### Sample size determination and sampling technique

For the first objective, the sample size was determined using the single population proportion formula, $$n = \frac{{\left( {Z{\alpha \mathord{\left/ {\vphantom {\alpha 2}} \right. \kern-0pt} 2}} \right)\,\,p\left( {1 - p} \right)}}{{d^{2} }}$$; assuming a 6.4% malaria prevalence (P) [21], $$Z_{{{\raise0.7ex\hbox{$\alpha $} \!\mathord{\left/ {\vphantom {\alpha 2}}\right.\kern-0pt} \!\lower0.7ex\hbox{$2$}}}} = 1.96$$ at 95% confidence level, a 2.5% margin of error (d), and including a 10% non-response rate, a minimum of 405 pregnant women were required. For the second objective, the sample size was determined by a double proportion formula using Epi Info version 7.2.2.6, considering different risk factors identified in the literature (maternal age, gravidity, maternal education, residence area, malnutrition, ABO blood group, ITN use, IRS status, presence of stagnant water, ANC follow-up status, and history of malaria in pregnancy). Among the predictors, the largest calculated minimum sample size was obtained for gravidity, assuming a 16.1% prevalence of malaria among primigravidae and a 6.5% prevalence of malaria among multigravidae from a study in northwest Ethiopia [23], at a 95% confidence level, 80% power, and an equal number of primigravidae and multigravidae. Including 10% non-response rate, a minimum of 418 parturient women were required. To increase representativeness, 526 pregnant women who were admitted to the delivery rooms of the health facilities for delivery or spontaneous abortion were recruited and included.

**Fig. 2:**
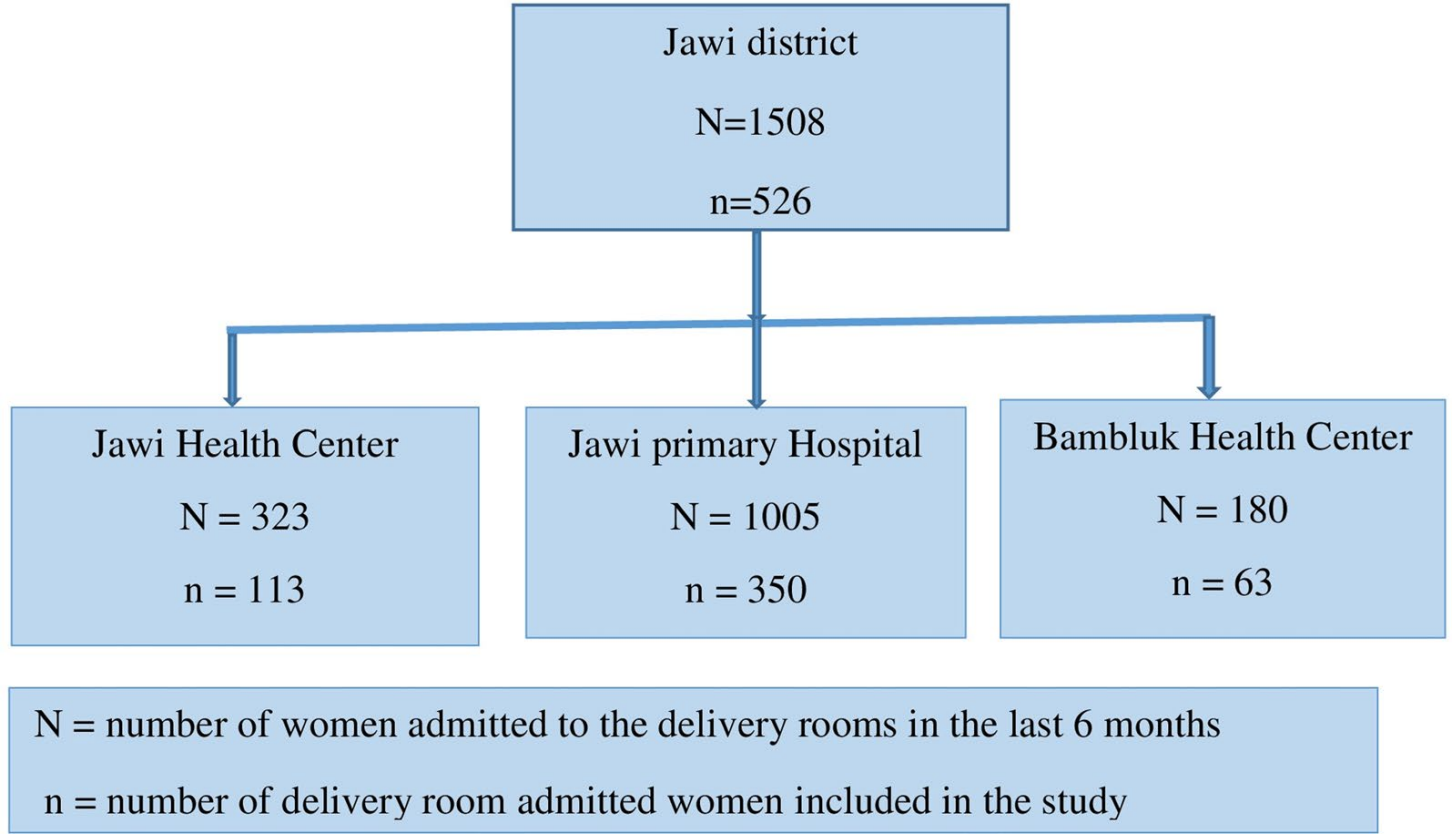
Sampling procedure of study participants from study health facilities

Probability proportionate to size sampling was used to determine the number of participants required from each health facility. The number of pregnant women from each health facility was determined based on the proportion of pregnant women who attended delivery rooms at each health facility in the last 6 months prior to the study. Accordingly, 350 women were included from the delivery rooms of Jawi Primary Hospital, 113 from Jawi Health Centre, and 63 from Bambluk Health Centre (Fig. 2).

Pregnant women who were admitted to the health facilities’ delivery rooms during the study period and were willing to participate in the study were eligible. Pregnant women who were HIV positive, hypertensive, diabetic, had multiple pregnancies, and had serious delivery complications were excluded. Those who met the eligibility criteria and consented to participate were included in the study.

### Socio-demographic and clinical data collection

Pretested structured questionnaires and checklists were used to collect socio-demographic, clinical, obstetric, and risk factor data from women who provided consent to participate in the study at the labour ward or postpartum. The questionnaire was initially prepared in English and later translated to the local languages (Amharic and Awungi) by native speakers of the languages. To ensure consistency, data collectors read and completed the Amharic or Awugni questionnaire for the participants, regardless of the participants’ literacy level.

The questionnaire included the women’s socio-demographic characteristics, malaria prevention practices, the presence of stagnant water in the household vicinity, and their estimated monthly household income. Similarly, information on obstetric and clinical profiles, including antenatal care follow-up, gravidity, parity, malaria symptoms, history of malaria during pregnancy, and usage of anti-malarial drugs were collected by interview and from medical follow-up cards. Gestational age was determined by the last menstrual period and the fundal height method in health centres, whereas the Mindray DP-50 digital ultrasound machine was used in the hospital. The temperature was recorded from the maternal armpit using a digital thermometer.

Mid-upper arm circumference (MUAC) was measured halfway between the tip of the shoulder (olecranon process) and the tip of the elbow (acromion process) to the nearest 0.1 cm (cm). An insertion-type MUAC tape that is non-elastic and non-stretchable was used to take the measurement. The measurement was taken at the mid-point on the relaxed left arm, without any clothing, and with optimal tape tension (not too loose or too tight) following the standard instructions and steps. Undernutrition was defined as MUAC less than 23 cm [24]. Maternal ABO and Rh blood types were determined by agglutination methods using commercial antisera.

### Blood specimen collection and malaria diagnosis

Maternal capillary blood was collected by finger pricking. Immediately after delivery, the maternal side of the placenta was cleaned with 0.9% normal saline, incised with a surgical blade, and blood was collected from the intervillous space with a syringe and transferred to a 0.5 millilitre Ethylene diamine tetraacetic acid (EDTA) tube. Similarly, the umbilical cord was clamped, cleaned with 0.9% saline (to prevent contamination with maternal blood), sectioned with a lancet and blood was collected with a syringe and transferred to an EDTA tube.

Malaria was diagnosed using light microscopy, rapid diagnostic tests (RDTs), and multiplex quantitative polymerase chain reaction (qPCR). The RDTs diagnosis of malaria was performed using the Abbott SD Bioline Malaria Ag *P.f/P.v*test kit (Standard Diagnostics, Inc., Republic of Korea) as per the manufacturer’s instructions. Thick and thin blood smears were prepared on a single microscope slide and allowed to air-dry at room temperature. Thin smears were fixed using absolute methanol and both the thick and thin smears were stained with a 10% Giemsa solution for 10 min, rinsed with tap water, and air dried [25]. Stained smears were diagnosed microscopically for the detection, identification, and quantification of malaria parasites. Both thick and thin smears were examined by two experienced laboratory technologists who were blinded to the RDT results. A blood film was declared negative after examination of at least 200 high-power microscope fields [25]. An expert microscopist who was blinded to the microscopy and RDT results re-checked all positive slides and 10% of negative slides to ensure quality.

The density of both sexual and asexual stages of *Plasmodium falciparum*, *Plasmodium vivax,* and *P. falciparum and P. vivax* mixed infections was estimated on thick film against 200 leucocytes, assuming a total white blood cell count of 8000/µl. The parasite density was then classified as low (below 1000 parasites per microlitre of blood), intermediate (1000–4999 parasites per microlitre of blood), and high (≥ 5000 parasites per microlitre of blood) [26].

### *Plasmodium* infection detection using real-time polymerase chain reaction

Finger-prick, placental and umbilical cord blood samples were spotted on Whatman filter papers, air-dried, packed in a ziplock containing desiccants, and transported by cold chain to the Aklilu Lemma Institute of Pathobiology (ALIPB) and stored at − 20 °C until analysed. A total of 1159 dry blood spots (DBSs) were collected from 372 delivering women and 43 abortion women. Accordingly, 12 peripheral blood DBSs were collected from 12 microscopic and/or RDT-positive aborting women, and 141 DBSs were collected from 47 delivering women who had microscopic and/or RDT-positive results in one or more of the compartments (47 peripheral, 47 placental, and 47 umbilical cord). The other 1006 DBSs were collected from 31 microscopic and/or RDT-negative aborting women (placental and umbilical cord blood was not available at this stage) and 325 delivering women (325 peripheral, 325 placental, and 325 umbilical cord). The real-time polymerase chain reaction assay was done at the Ethiopian Public Health Institute.

Genomic DNA (gDNA) extraction was performed using the Geneius™ Micro gDNA Extraction Kit (Geneaid Biotech Ltd., Taiwan). Briefly, 3 mm-diameter circles of DBS were punched out and processed following the manufacturer’s instructions in 1.5 ml Eppendorf tubes. The resulted DNA was eluted with 100 µl volume of elution buffer and stored at − 20 °C until assayed.

**Fig. 3:**
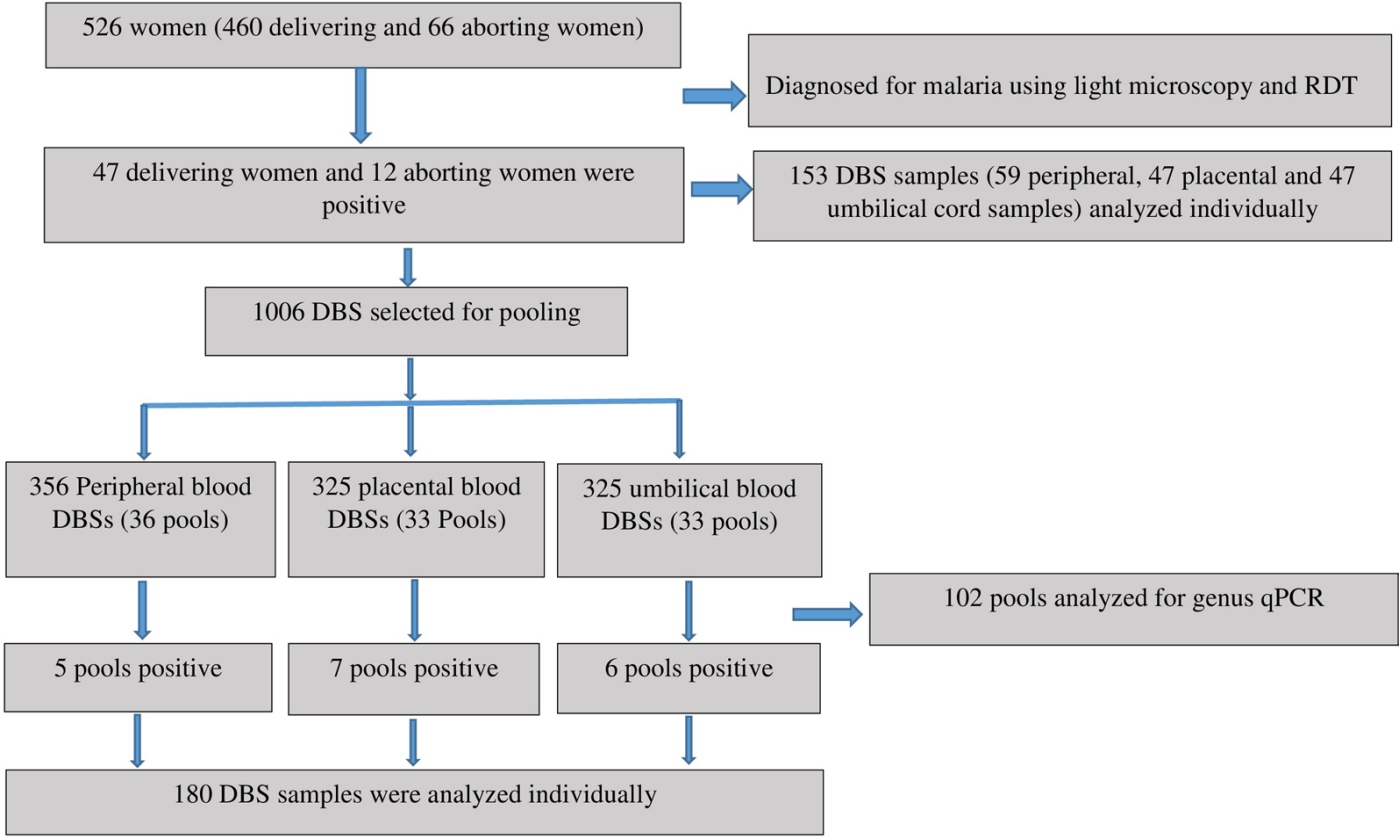
Flow chart showing pooling and analysis procedures of dry blood spots in quantitative polymerase chain reaction

All microscopy and/or RDT-positive samples were extracted and analysed individually. For microscopy and RDT-negative samples, malaria prevalence was estimated using pooled DBS sample extraction and analysis with slight modifications as described by Zhou et al*.* [27] (Fig. 3).

In short, ten-punched out 3 mm-diameter circles were pooled together in 2 ml Eppendorf tubes and incubated overnight with lyse buffer and proteinase K solution to ensure proper lysis, and then DNA extraction was performed following the normal procedures. The extracted DNA was tested for the *Plasmodium* genus using multiplex real-time PCR (qPCR). Samples in genus qPCR-negative pools were taken as negative. For any pool with a positive genus qPCR, individual DBSs were extracted following the protocol, tested for genus-specific qPCR and then species-specific qPCR assays were performed.

The DNA amplification and detection were performed by the QuantStudio 5 Real Time PCR system (Applied Biosystems) using the TaqMan fluorescence assay in a final volume of 10 μl in two rounds. In the first run, all samples were tested by multiplexing pan-*Plasmodium*-specific and *P. falciparum*-specific primers; whereas *P. falciparum* and *P. vivax*-specific primers were multiplexed during the second run, as described in Belachew et al*.* [28] (Table 1).

**Table 1:** Primers and probes used for detection of *Plasmodium* species in pregnancy, northwest Ethiopia, 2022

Target Gene	Sequence	Fluorophores
Pspp18S F	GCT CTT TCT TGA TTT CTT GGA TG	–
Pspp18S R	AGC AGG TTA AGA TCT CG TTC G	–
Pspp18S Cy5	ATG GCC GTT TTT AGT TCG TG	Cy5-BHQ2
HsRNaseP F	AGA TTT GGA CCT GCG AGC G	–
HsRNaseP R	GAG CGG CTG TCT CCA CAA GT	–
HsRNaseP YYE	TTC TGA CCT GAA GGC TCT GCG CG	YYE-BHQ1
PfvarATS F	CCCATACACAACCAAYTGGA	–
PfvarATS R	TTCGCACATATCTCTATGTCTATCT	–
PfvarATS FAM	TRTTCCATAAATGGT	Fluorescein
Pv18S F	ACTAGGCTTTGGATGAAAGATTTTA	–
Pspp18S R	AACCCAAAGACTTTGATTTCTCATAA	–
Pv18S probe	GAATTTTCTCTTCGGAGTTTAT	Cy5-BHQ2

The PCR amplifications were conducted with the following thermal cycling conditions: 95 °C for 1 min, followed by 45 cycles of 95 °C for 15 s and 57 °C for 45 s for the first PCR run; and 95 °C for 1 min, followed by 45 cycles of 95 °C for 15 s and 53 °C for 45 s for the second run. In each experiment, the 3D7 DNA standard was run as a positive control and nuclease free water as a negative control. For the PCR run, Ct values of 25.0 to 30.0 for the positive control and Ct values < 30.0 for HsRNaseP to all samples taken as qualified run. Samples with Ct values between 12 and 40 and sigmoidal shape amplification curve were considered positive.

### Operational definitions

*Plasmodium* infection**:** having a positive RDT and/or detection of malaria parasites using light microscopy and/or real-time qPCR.

Malaria at parturiency: Parasitaemia detected at least in one of the three blood specimen types (capillary, placental, or umbilical cord blood).

Congenital malaria: *Plasmodium* infection in umbilical cord blood at delivery.

Malaria history in pregnancy**:** history of malaria infection during pregnancy recorded in the maternal treatment card.

Asymptomatic *Plasmodium* infection**:** *Plasmodium* infection in the absence of malaria symptoms, a history of fever within the past 48 h, and an axillary temperature < 37.5 °C.

Symptomatic *Plasmodium* infection**:** *Plasmodium* infection from women who exhibited at least one of the signs and/or symptoms of malaria like axillary temperature ≥ 37.5 °C, fever, joint pain, malaise, vomiting, chills, and headache during delivery room admission or within the past 48 h.

Submicroscopic *Plasmodium* infection: Having positive RDT and/or detection of malaria parasites by real-time qPCR but not by microscopy.

Subpatent infection**:** Malaria parasites not detected by microscopy and negative by malarial RDT but detected by real time qPCR.

Multigravida: A woman who is pregnant for the second time and above.

Multipara: A woman who delivered for the second time and more.

### Statistical analysis

Data was double entered into Epi data 3.1 software to reduce clerical errors and inconsistencies. The entered data was exported to Statistical Package for Social Sciences (SPSS) version 25 statistical software (IBM Corp., New York, USA), cleaned, and analysed. *Plasmodium* infection was defined as having a positive RDT and/or detection of malaria parasites using light microscopy and/or real-time qPCR. The prevalence of *Plasmodium* species infections in peripheral, placental, and cord blood samples was estimated by dividing the number of women diagnosed positive for *Plasmodium* infection by the total number of pregnant women examined for peripheral, placental, and umbilical cord infections, respectively.

Bivariable and multivariable logistic regression models were used to identify independent predictors of malaria. Only variables that showed statistical significance at p < 0.05 in the bivariable logistic regression analysis and those known to be associated with malaria based on previous studies were included in the multivariable logistic regression model. Multicollinearity among predictors was tested by using the variance inflation factor (VIF < 10). For the multivariable logistic regression analysis, the standard (Enter) method was used for variable selection. The model’s fitness was checked using the Hosmer and Lemeshow test (p > 0.05). The Odds ratio (OR) with a 95% confidence interval (CI) was used to measure the strength of the statistical association. A p-value < 0.05 was used to indicate statistical significance.

## Results

### Baseline characteristics of parturient women

A total of 526 delivery room-admitted consented pregnant women were examined for *Plasmodium* infections, among which 87.5% (n = 460) were admitted for delivery and 12.5% (n = 66) for spontaneous abortion. The median (interquartile range) age of the women was 25 (8) years, and the majority (80%) were young adults aged 20–34 years. About 48.1% of the women resided in rural areas, 47.7% were illiterate, and 51.9% were engaged in farming. More than 55% of them were primigravidae or secundigravidae, and 63.7% had at least one ANC follow-up during the current pregnancy. Moreover, only 29.8% of them reported that they slept under ITNs on the previous night of the delivery room admission, and 46.4% of the households did not sprayed with IRS within the last 12 months. Among the women, 40.9% had the O blood group phenotype, and 2.9% had symptoms of malaria during or within 48 h of admission to the delivery rooms (Table 2).

**Table 2:** Baseline characteristics of parturient women in Jawi district health, northwest Ethiopia, 2022

Characteristics	Category	n (%)
Age category in years	<20	48 (9.1)
	20–34	421 (80.0)
	≥ 35	57 (10.8)
Residence	Urban	273(51.9)
	Rural	253(48.1)
Marital status	Single	33 (6.3)
	Married	493 (93.7)
Education status	Illiterate	251 (47.7)
	Primary education	154 (29.3)
	Secondary education	78 (14.8)
	College and above	43 (8.2)
Family size	< 5	341 (64.8)
	≥ 5	185 (35.2)
Gravidity	Primigravida	141 (26.8)
	Secundigravida	151 (28.7)
	Multigravida	234 (44.5)
ABO blood group	A	166 (31.6)
	B	121 (23)
	AB	24 (4.6)
	O	215 (40.9)
Antenatal care follow up	No follow up	191 (36.3)
	Partial (1–3)	198 (37.16)
	Full follow up(4)	137 (26)
Measurement of upper arm circumstance (MUAC)	< 23 cm	127 (24.1)
	≥ 23 cm	399 (75.9)
Slept under ITN the night before admission to delivery room	No	369 (70.2)
	Yes	157(29.8)
IRS within the last 1 year	No	244 (46.4)
	Yes	282 (53.6)
Presence of stagnant water in the house vicinity	No	468 (89)
	Yes	58 (11)
History of malaria infection during pregnancy	Yes	73 (13.9)
	No	453 (86.1)
Symptoms of malaria at parturiency	Yes	15 (2.9)
	No	511 (97.1)

### Prevalence of *Plasmodium* infections among parturient women

Overall, 14.3% (75/526, 95% CI 11.4–17.5%) of the admitted parturient women had *Plasmodium* infection. Among the parturient women, 12.2% (64/526, 95% CI 9.5–15.3%) had peripheral malaria and 10.9% (50/460, 95% CI 8.2–14.1) had placental malaria. About 3.7% (17/460, 95% CI 2.3–6.1) of the newborns had congenital malaria. All congenitally infected newborns were from mothers who had both peripheral and placental malaria. However, twenty women had discordant results between peripheral and placental blood specimens (nine had peripheral *Plasmodium* infection only and eleven had placental *Plasmodium* infection only). *Plasmodium falciparum* accounted for 76.6% of peripheral, 82% of placental, and 88.2% of congenital malaria infections. Asymptomatic malaria was 11.4% (58/511) in peripheral blood and 10.3% (46/448) in placental blood specimens, which accounted for 90.6% of the peripheral and 92% of the placental *Plasmodium* infections. On the other hand, 40% of the symptomatic parturient women had peripheral malaria and 33.3% had placental malaria, which accounted for 9.4% of the peripheral and 8% of the placental malaria cases, respectively (Table 3).

**Table 3:** *Plasmodium* infections among parturient women in Jawi district, northwest Ethiopia, 2022

Malaria in pregnancy	Malaria, % (n/N)
**Peripheral malaria** (N = 526)	12.2% (64/526)
Plasmodium species	
*Plasmodium falciparum*	9.3% (49/526)
*Plasmodium vivax*	2.1% (11/526)
Mixed (*P.falciparum* and *P.vivax*)	0.8% (4/526)
Asymptomatic peripheral malaria	11.4% (58/511)
Symptomatic malaria	40% (6/15)
Submicroscopic	3.6% (19/526)
Microscopic	8.6% (45/526)
Parasite density in microscopic cases	
Median (IQR) parasite density	5160 (14,420)
Low	51.1% (23/45)
Intermediate	28.9% (13/45)
High	20% (9/45)
**Placental malaria** (N = 460)	10.9% (50/460)
*Plasmodium* species	
*Plasmodium falciparum*	8.9% (41/460)
*Plasmodium vivax*	1.3% (6/460)
Mixed(*P.falciparum* and *P.vivax*)	0.7% (3/460)
Asymptomatic placental malaria (N = 448)	10.3% (46/448)
Symptomatic placental malaria(N = 12)	33.3% (4/12)
Submicroscopic	4.8% (22/460)
Microscopic	6.1% (28/460)
Parasite density in microscopic cases, (n = 28)	
Median (IQR) parasite density	17,000 (5150)
Low	35.7% (10/28)
Intermediate	39.3% (11/28)
High	25% (7/28)
**Congenital malaria** (N = 460)	3.7% (17/460)
Plasmodium species	
*Plasmodium falciparum*	3.3% (15/17)
*Plasmodium vivax*	0.4% (2/17)
Congenital malaria from asymptomatic women (N = 448)	3.3% (15/448)
Congenital malaria from symptomatic women(N = 12)	16.7% (2/12)
Submicroscopic	3.5% (16/460)
Microscopic	0.2% (1/460)

### Risk factors of *Plasmodium* infection in pregnancy

In multivariable logistic regression analyses, illiterate parturient women had more than a twofold increased odds of peripheral malaria than the literates (AOR = 2.03, 95% CI 1.11–3.74). The primigravidae were 1.88 times more likely to acquire *Plasmodium* infection than their multigravid counterparts (AOR = 1.88, 95% CI 1.01–3.49). Similarly, parturient women who did not have ANC follow-up during pregnancy had more than a double increased risk of malaria at the end of pregnancy compared to women who had ANC follow-up (AOR = 2.28, 95% CI 1.04, 5.03). Moreover, women who had a history of symptomatic malaria during pregnancy had a more than fourfold increased risk of malaria at delivery (AOR = 4.2, 95% CI 2.32–7.59) (Table 4). In this study, placental malaria was significantly associated with the O blood group, revealing more than a double risk of placental malaria among blood group O women than the non-O ABO blood groups (COR = 2.25, 95% CI 1.24–4.08). When the association of placental malaria with the ABO blood group was analysed by parity, group O primiparae had a 5.27 times higher risk of placental malaria compared to their non-O counterparts (COR = 5.27, 95% CI 1.92–14.4). However, the ABO blood group did not have a significant association with placental malaria among the multiparae (COR = 1.17, 95% CI 0.52–2.62) (Table 5).

**Table 4:** Factors associated with malaria among parturient women in Jawi district, northwest Ethiopia, 2022

Variables		Malaria in pregnancy				
		Yes, n (%)	No, n (%)	COR [95% CI]	P-value	AOR [95% CI]
Age category	< 20 years	12 (25.0)	36 (75.0)	2.2 [1.1,4.4]	0.029	1.29 [0.55, 3.01]
	≥ 20 years	63 (13.2)	415 (86.8)	1		1
Educational status	Illiterate	49 (19.5)	202 (80.5)	2.32 [1.4,3.9]	0.001	**2.03 [1.11, 3.74]**
	Literate	26 (9.5)	249 (90.5)	1		1
Residence area	Urban	29 (10.6)	244 (80.4)	1		1
	Rural	46 (18.2)	207 (81.8)	1.9 [1.1, 3.1]	0.014	1.12 [0.63, 2.02]
Gravidity	Primigravida	28 (19.9)	113 (80.1)	1.8 [1.1, 3.0]	0.028	**1.88 [1.01, 3.49]**
	Multigravida	47 (12.2)	338 (87.8)	1		1
Slept under ITN the night before survey	No	62 (16.8)	307 (83.2)	2.2 [1.2, 4.2]	0.012	1.82 [0.93, 3.57]
	Yes	13 (8.3)	144 (91.7)	1		1
House spray	Yes	36 (12.8)	246 (87.2)	1		
	No	39 (16.0)	205 (84.0)	1.3 [0.8, 2.1]	0.293	
Stagnant water	No	12 (20.7)	46 (79.3)	1.7 [0.8, 3,3]	0.141	
	Yes	63 (13.5)	405 (86.5)	1		
History of Malaria in pregnancy	No	28 (38.4)	45 (61.6)	5.4 [3.1, 9.4]	0.001	**4.2 [2.32, 7.59]**
	Yes	47 (10.4)	406 (89.6)	1		1
ANC follow up	No	39 (20.4)	152 (79.6)	3.3 [1.6, 6.8]	0.002	**2.28 [1.04, 5.03]**
	Partial (≤ 3)	26 (13.1)	172 (86.9)	1.9 [0.9, 4.1]	0.095	1.41 [0.62, 3.21]
	Full (4)	10 (7.3)	127 (92.7)	1		1
MUAC	< 23	45 (23.6)	354 (76.4)	2.4 [1.5, 4.1]	0.001	1.65 [0.94, 2.92]
	≥ 23	30 (11.3)	97 (88.7)	1		1
ABO blood group	Non-O	39 (12.5)	272 (87.5)	1		
	O	36 (16.7)	179 (83.3)	1.4 [0.9, 2.3]	0.177	

Bold values in the AOR[95% CI) column indicate statistical significance in multivariable logistic regression analysis

**Table 5:** Association of placental malaria and ABO blood group overall and by parity in Jawi district, northwest Ethiopia, 2022

			Placental malaria		COR [95% CI]	P-value
			Yes, n (%)	No, n (%)		
Overall (n = 460)	ABO phenotype	Non-O	21 (7.6)	254 (92.4)	1	0.008
		O	29 (15.7)	156 (84.3)	2.25 [1.24, 4.08]	
Primiparae, (n = 126)	ABO phenotype	Non-O	6 (8.5)	65 (91.5)	1	0.002
		O	18 (32.7)	37 (67.3)	5.27 [1.92,14.4]	
Multiparae (n = 334)	ABO phenotype	Non-O	15 (7.4)	189 (92.6)	1	0.688
		O	11 (8.5)	119 (91.5)	1.17 [0.52,2.62]	

## Discussion

In this study, 14.3% of the parturients admitted to the delivery rooms in three health facilities in Jawi district, northwest Ethiopia, had *Plasmodium* infections, and the majority of the infections were due to *P. falciparum*. About 12.2% of the parturient women had peripheral malaria, 10.9% of the delivered women had placental malaria, and 3.7% of the newborns had congenital malaria. Maternal illiteracy, primigravidity, lack of ANC follow-up, and history of malaria during pregnancy were independent predictors of malaria at the end of pregnancy. Unlike the multiparae, blood group O primiparae had a significantly increased risk of placental malaria compared to the non-O ABO blood primiparae. Such findings of malaria in pregnancy could have a substantial impact, as malaria in pregnancy is associated with poor maternal, fetal, and childhood outcomes [29].

The prevalence of *Plasmodium* infection in pregnancy in the current study was comparable with a study in southern Ethiopia [20], but relatively higher than studies in northwest and southwest Ethiopia [12, 21]. On the other hand, higher prevalences of malaria were reported in Papua New Guinea [30], Sudan [31], and Colombia [32]. In line with this study’s findings, the study conducted in selected unstable malaria transmission areas in Ethiopia reported that the majority of peripheral and placental infections were due to *P*. *falciparum* [12]. On the other hand, the study in southern Ethiopia showed the dominance of *P. vivax* in pregnancy [20]. Differences in malaria transmission intensity and *Plasmodium* species distribution in the areas, the socio-demographic characteristics and conditions of pregnant women (presence of malaria symptoms, nutritional status, and practice of malaria preventive methods), and the diagnostic efficiency of the malaria detection methods used could be possible explanations for the variation in malaria prevalence and *Plasmodium* species distribution between the current study and previous studies.

In this study, all congenital *Plasmodium* infections were from women who had both peripheral and placental *Plasmodium* infections. However, nine women had a peripheral *Plasmodium* infection only, and eleven women had a placental *Plasmodium* infection only. This finding was consistent with previous studies, which reported different levels of discordance [20, 33]. Peripheral parasitaemia without placental *Plasmodium* infection might be due to early infections (particularly in low parasitaemia cases), a lack of VAR2CSA protein expression on infected red blood cells [34], and the presence of antibodies to VAR2CSA that might inhibit the binding of infected RBCs to CSA in the placenta [35].On the other hand, placental infection without peripheral parasitaemia might be a result of peripheral parasitaemia clearance but not placental parasitaemia due to evasion by sequestration or due to previous effective treatment of malaria that might have cleared peripheral parasitaemia but not placental parasitaemia.

Studies have shown that susceptibility to malaria and the severity of infections during pregnancy differ with the intensity of malaria transmission in the area, the level of exposure, and the anti-malarial immunity acquired by the pregnant woman [13, 36]. Thus, young and primigravid women are highly susceptible and experience severe illness in low and unstable transmission areas, whereas most infections are asymptomatic among older and multigravid women in high and stable transmission areas [12, 13]. In the current study, more than 90% of *Plasmodium* infections were asymptomatic and more than 30% were submicroscopic. *Plasmodium* infections were significantly higher among adolescent women (< 20 years) and independently predicted by primigravidity. In line with the present study, studies in Papua New Guinea and Uganda reported that primigravid women had an increased risk of placental and peripheral malaria compared to multigravid women [30, 37]. Since only symptomatic cases are diagnosed for malaria in Ethiopia [16], this study revealed that the majority of malaria cases in the study area would go undiagnosed, which poses deleterious effects on maternal health and birth outcomes [38]. Moreover, these malaria parasites sequester in the placenta and cause long-lasting infections that could be a hub of malaria transmission in the community, thus hindering malaria elimination efforts in the country.

Pregnant women in Ethiopia are targeted during malaria prevention and control activities. Malaria preventive and curative interventions are also provided on the ANC platform [17]. In the current study, about half of the women were illiterate and more than a third did not have any ANC follow-up. The study showed that illiteracy and lack of ANC follow-up are independent predictors of malaria in pregnancy. In line with these findings, a study in Ghana and Uganda reported lower maternal education status as a predictor of malaria in pregnancy [39, 40]. This might be due to the fact that illiterate women are less aware of the effects of malaria in pregnancy and thus may not practice the necessary malaria prevention and control methods. Thus, the lower malaria risk of women who had ANC follow-up in the current study might be a result of the malaria preventive awareness and curative interventions provided on the ANC platform, such as health education, the provision of ITNs, and the diagnosis and treatment of symptomatic mothers.

In the current study, there was a significant association between a history of malaria during pregnancy and *Plasmodium* infections at delivery, despite treatment of women with anti-malarial drugs upon diagnosis. Since only symptomatic cases are screened for malaria and light microscopy is the standard of malaria diagnosis in health centres and hospitals in Ethiopia [16], this data should be interpreted with caution because it did not assess the impact of asymptomatic and submicroscopic infections. The association of malaria in pregnancy with malaria at delivery despite treatment might be associated with the persistence of the previous infection during pregnancy due to improper drug use, drug resistance, or re-infection. A Ugandan study reported the absence of placental infections among women who did not have malaria in pregnancy and the association of both microscopic and submicroscopic infections in pregnancy with placental infections, emphasizing timing of infection, frequency, and parasite densities as risk factors for placental infections [41]. Similarly, a study in Angola showed that a self-reported history of malaria infection during pregnancy was a significant predictor of peripheral, placental, and congenital malaria infections at delivery [42].

In this study, a significantly increased risk of placental malaria was observed among blood group O-women than the non-O phenotypes. In the subgroup analysis, blood group O primiparae had about a fivefold higher risk of placental malaria than the non-O blood groups, which was not observed among multigravid women. The higher risk of placental *Plasmodium* infection among blood group O primiparae is consistent with studies in Gambia [43], Sudan [44], and Malawi [45]. The susceptibility difference and parity-specific associations of ABO phenotypes with placental malaria might be due to the effect of the ABO phenotype on the level of the proteoglycan thrombomodulin, which is present in the placenta and mediates the binding of malaria-infected RBCs with CSA [46, 47]. Moreover, the more attractive nature of pregnant women for malaria mosquitoes [48] and a preferential feeding of *Anopheles gambiae* mosquitoes to blood group O [49] could be the possible reasons for the frequent occurrence of malaria among O-blood group mothers.

Taken together, this study provided important information that was limited in Ethiopia and the first in the study area, from a relatively representative sample size, using different diagnostic methods (light microscopy, RDTs, and qPCR), and considering diverse factors compared to previous studies in Ethiopia, such as the ABO blood group. However, the findings of the current study should be considered with their limitations. This study did not use histopathological techniques for the diagnosis of placental malaria which could underestimate the prevalence. The qPCR diagnosis of malaria was not done for all study participants. Moreover, it would be better if malaria was screened from newborn peripheral blood in addition to umbilical cord blood for screening of congenital *Plasmodium* infections.

## Conclusion

Overall, this study showed that *Plasmodium* infections, the majority of which were asymptomatic cases, were prevalent among parturients in northwest Ethiopia. Maternal illiteracy, lack of ANC follow-up, primigravidity, and malaria infection during pregnancy were significantly associated with malaria at the end of pregnancy. Moreover, blood group O was associated with a higher risk of placental malaria, particularly in primiparae. Thus, promotion of a healthy pregnancy through ANC follow-up, strengthening malaria prevention and control practices, and routine screening of malaria in asymptomatic pregnant women are suggested to reduce the burden of malaria in pregnancy.

## Data Availability

All data generated or analysed during this study are included in this published article.

## Abbreviations

ANC: Antenatal care
AOR: Adjusted odds ratio
BHC: Bambluk health centre
CSA: Chondroitin sulfate A
CI: Confidence interval
COR: Crude odds ration
Ct: Cycle threshold
DBSs: Dry blood spots
gDNA: Genomic deoxyribonucleic acid
INTs: Insecticide-treated nets
IRS: Indoor residual spray
JHC: Jawi health centre
JPH: Jawi Primary Hospital
OR: Odds ratio
qPCR: Quantitative polymerase chain reaction
RDT: Rapid diagnostic tests
SD: Standard deviation
VAR2CSA: Variant surface antigen 2-chondroitin sulfate A

## References

[1] WHO (2022). World malaria report.

[2] Ethiopian Public Health Institute (2016). Ethiopia national malaria indicator survey 2015.

[3] Mellor AL, Munn DH (2001). Extinguishing maternal immune responses during pregnancy: implications for immunosuppression. Semin Immunol.

[4] Carmona-Fonseca J, Cardona-Arias JA (2022). Placental malaria caused by *Plasmodium*
*vivax* or P falciparum in Colombia: Histopathology and mediators in placental processes. PLoS One..

[5] Beeson JG, Amin N, Kanjala M, Rogerson SJ (2002). Selective accumulation of mature asexual stages of *Plasmodium falciparum*-infected erythrocytes in the placenta. Infect Immun.

[6] Salanti A, Dahlback M, Turner L, Nielsen MA, Barfod L, Magistrado P (2004). Evidence for the involvement of VAR2CSA in pregnancy-associated malaria. J Exp Med.

[7] Enweronu-Laryea CC, Adjei GO, Mensah B, Duah N, Quashie NB (2013). Prevalence of congenital malaria in high-risk Ghanaian newborns: a cross-sectional study. Malar J.

[8] Walker PGT, ter Kuile FO, Garske T, Menendez C, Ghani AC (2014). Estimated risk of placental infection and low birthweight attributable to *Plasmodium falciparum* malaria in Africa in 2010: a modelling study. Lancet Glob Health.

[9] Bilal JA, Malik EE, Al-Nafeesah A, Adam I (2020). Global prevalence of congenital malaria: a systematic review and meta-analysis. Eur J Obstet Gynecol Reprod Biol.

[10] Rogerson SJ, Mwapasa V, Meshnick SR (2007). Malaria in pregnancy: linking immunity and pathogenesis to prevention. Am J Trop Med Hyg.

[11] Desai M, ter Kuile FO, Nosten F, McGready R, Asamoa K, Brabin B (2007). Epidemiology and burden of malaria in pregnancy. Lancet Infect Dis.

[12] Newman RD, Nahlen BL, Hailemariam A, Jimma D, Degifie A, Kebede D (2003). Burden of malaria during pregnancy in areas of stable and unstable transmission in Ethiopia during a non epidemic year. J Infect Dis.

[13] Tagbor H, Bruce J, Browne E, Greenwood B, Chandramohan D (2008). Malaria in pregnancy in an area of stable and intense transmission: is it asymptomatic?. Trop Med Int Health.

[14] Boudová S, Cohee LM, Kalilani-Phiri L, Thesing PC, Kamiza S, Muehlenbachs A (2014). Pregnant women are a reservoir of malaria transmission in Blantyre. Malawi Malar J.

[15] WHO (2004). A strategic framework for malaria prevention and control during pregnancy in the African Region.

[16] Ministry of Health-Ethiopia (2020). National malaria elimination strategic plan: 2021–2025.

[17] Federal Democratic Republic of Ethiopia Ministry of Health (2010). Management protocol on selected obstetrics topics.

[18] Gultie T, Ayele G, Tariku B, Kondale M, Zerdo Z, Merdekiyos B (2020). Trend of declining bed net utilization among pregnant women in Ethiopia: new data from the Arba minch health and demographic surveillance system, 2010–2016. Malar J.

[19] Central Statistical Agency, ICF. Ethiopia demographic and health survey 2016. Addis Ababa. 2017.

[20] Solomon A, Kahase D, Alemayhu M (2020). Prevalence of placental malaria among asymptomatic pregnant women in Wolkite health center, Gurage zone, Southern Ethiopia. Trop Dis Travel Med Vaccines.

[21] Limenih A, Gelaye W, Alemu G (2021). Prevalence of malaria and associated factors among delivering mothers in Northwest Ethiopia. Biomed Res Int.

[22] Amare A, Eshetu T, Lemma W (2022). Dry-season transmission and determinants of *Plasmodium* infections in Jawi district, northwest Ethiopia. Malar J.

[23] Tilahun A, Yimer M, Gelaye W, Tegegne B (2020). Prevalence of asymptomatic Plasmodium species infection and associated factors among pregnant women attending antenatal care at Fendeka town health facilities, Jawi District, North west Ethiopia: a cross-sectional study. PLoS ONE.

[24] Ververs MT, Antierens A, Sackl A, Staderini N, Captier V (2013). Which anthropometric indicators identify a pregnant woman as acutely malnourished and predict adverse birth outcomes in the humanitarian context?. PLoS Curr.

[25] WHO (2015). Methods Manual Microscopy for the detection, identification and quantification of malaria parasites on stained thick and thin blood films: procedure.

[26] Abebe A, Menard D, Dugassa S, Assefa A, Juliano JJ, Lo E (2023). Significant number of *Plasmodium* vivax mono-infections by PCR misidentified as mixed infections (P vivax/P falciparum) by microscopy and rapid diagnostic tests: malaria diagnostic challenges in Ethiopia. Malar J.

[27] Zhou Z, Mitchell RM, Gutman J, Wiegand RE, Mwandama DA, Mathanga DP (2014). Pooled PCR testing strategy and prevalence estimation of submicroscopic infections using Bayesian latent class models in pregnant women receiving intermittent preventive treatment at Machinga District Hospital, Malawi, 2010. Malar J.

[28] Belachew M, Wolde M, Nega D, Gidey B, Negash L, Assefa A (2022). Evaluating performance of multiplex real time PCR for the diagnosis of malaria at elimination targeted low transmission settings of Ethiopia. Malar J.

[29] Walther B, Miles DJ, Crozier S, Waight P, Palmero MS, Ojuola O (2014). Placental malaria is associated with reduced early life weight development of affected children independent of low birth weight. Malar J.

[30] Lufele E, Umbers A, Ordi J, Ome-Kaius M, Wangnapi R, Unger H (2017). Risk factors and pregnancy outcomes associated with placental malaria in a prospective cohort of Papua New Guinean women. Malar J.

[31] Omer SA, Idress HE, Adam I, Abdelrahim M, Noureldein AN, Abdelrazig AM (2017). Placental malaria and its effect on pregnancy outcomes in Sudanese women from Blue Nile State. Malar J.

[32] Arango EM, Samuel R, Agudelo OM, Carmona-Fonseca J, Maestre A, Yanow SK (2013). Molecular detection of malaria at delivery reveals a high frequency of submicroscopic infections and associated placental damage in pregnant women from northwest Colombia. Am J Trop Med Hyg.

[33] Ezebialu IU, Eke AC, Ezeagwuna DA, Nwachukwu CE, Ifediata F, Ezebialu CU (2012). Prevalence, pattern, and determinants of placental malaria in a population of southeastern Nigerian parturients. Int J Infect Dis.

[34] Cohee LM, Kalilani-Phiri L, Mawindo P, Joshi S, Adams M, Kenefic L (2016). Parasite dynamics in the peripheral blood and the placenta during pregnancy-associated malaria infection. Malar J.

[35] Babakhanyan A, Fang R, Wey A, Salanti A, Sama G, Efundem C (2015). Comparison of the specificity of antibodies to VAR2CSA in Cameroonian multigravidae with and without placental malaria: a retrospective case-control study. Malar J.

[36] Tran EE, Cheeks ML, Kakuru A, Muhindo MK, Natureeba P, Nakalembe M (2020). The impact of gravidity, symptomatology and timing of infection on placental malaria. Malar J.

[37] De Beaudrap P, Turyakira E, White LJ, Nabasumba C, Tumwebaze B, Muehlenbachs A (2013). Impact of malaria during pregnancy on pregnancy outcomes in a Ugandan prospective cohort with intensive malaria screening and prompt treatment. Malar J.

[38] Cottrell G, Moussiliou A, Luty AJ, Cot M, Fievet N, Massougbodji A (2015). Submicroscopic *Plasmodium falciparum* infections are associated with maternal anemia, premature births, and low birth weight. Clin Infect Dis.

[39] Ahenkorah B, Nsiah K, Baffoe P, Ofosu W, Gyasi C, Owiredu EW (2020). Parasitic infections among pregnant women at first antenatal care visit in northern Ghana: a study of prevalence and associated factors. PLoS ONE.

[40] Okiring J, Olwoch P, Kakuru A, Okou J, Ochokoru H, Ochieng TA (2019). Household and maternal risk factors for malaria in pregnancy in a highly endemic area of Uganda: a prospective cohort study. Malar J.

[41] Briggs J, Ategeka J, Kajubi R, Ochieng T, Kakuru A, Ssemanda C (2019). Impact of microscopic and submicroscopic parasitemia during pregnancy on placental malaria in a high-transmission setting in Uganda. J Infect Dis.

[42] Valente B, do Campos PA, Rosario VE, Varandas L, Silveira H (2011). Prevalence and risk factors of *Plasmodium falciparum* infections in pregnant women of Luanda Angola. Trop Med Int Health..

[43] Loscertales MP, Brabin BJ (2006). ABO phenotypes and malaria related outcomes in mothers and babies in The Gambia: a role for histo-blood groups in placental malaria?. Malar J.

[44] Adam I, Babiker S, Mohmmed AA, Salih MM, Prins MH, Zaki ZM (2007). ABO blood group system and placental malaria in an area of unstable malaria transmission in eastern Sudan. Malar J.

[45] Senga E, Loscertales MP, Makwakwa KE, Liomba GN, Dzamalala C, Kazembe PN (2007). ABO blood group phenotypes influence parity specific immunity to *Plasmodium falciparum* malaria in Malawian women. Malar J.

[46] Rogerson SJ, Novakovic S, Cooke BM, Brown GV (1997). *Plasmodium falciparum*-infected erythrocytes adhere to the proteoglycan thrombomodulin in static and flow-based systems. Exp Parasitol.

[47] Blann AD, Daly RJ, Amiral J (1996). The influence of age, gender and ABO blood group on soluble endothelial cell markers and adhesion molecules. Br J Haematol.

[48] Lindsay S, Ansell J, Selman C, Cox V, Hamilton K, Walraven G (2000). Effect of pregnancy on exposure to malaria mosquitoes. Lancet.

[49] Wood CS (1974). Preferential Feeding of *Anopheles gambiae* mosquitoes on human subjects of blood group O: a relationship between the ABO polymorphism and malaria vectors. Hum Biol.

